# A mathematical model shows macrophages delay *Staphylococcus aureus* replication, but limitations in microbicidal capacity restrict bacterial clearance

**DOI:** 10.1016/j.jtbi.2020.110256

**Published:** 2020-07-21

**Authors:** Alex Best, Jamil Jubrail, Mike Boots, David Dockrell, Helen Marriott

**Affiliations:** a School of Mathematics & Statistics, University of Sheffield, Sheffield, S3 7RH, UK; b Medical School, Dept of Infection Immunity and Cardiovascular Disease, University of Sheffield, Sheffield, S10 2RX, UK; c Centre for Inflammation Research, Queen's Medical Research Institute, Edinburgh BioQuarter, Edinburgh, EH16 4TJ, UK; d Integrative Biology, University of California Berkeley, Berkeley, CA 94720-3140, USA; e Biosciences, College of Life & Environmental Sciences, University of Exeter Cornwall Campus, Penryn, TR10 9EZ, UK; f Department of Infection Medicine and MRC Centre for Inflammation Research, University of Edinburgh

**Keywords:** *S. aureus*, Macrophage, Infection, Phagocytosis

## Abstract

*S. aureus* is a leading cause of bacterial infection. Macrophages, the first line of defence in the human immune response, phagocytose and kill *S. aureus* but the pathogen can evade these responses. Therefore, the exact role of macrophages is incompletely defined. We develop a mathematical model of macrophage - *S. aureus* dynamics, built on recent experimental data. We demonstrate that, while macrophages may not clear infection, they significantly delay its growth and potentially buy time for recruitment of further cells. We find that macrophage killing is a major obstacle to controlling infection and ingestion capacity also limits the response. We find bistability such that the infection can be limited at low doses. Our combination of experimental data, mathematical analysis and model fitting provide important insights in to the early stages of *S. aureus* infections, showing macrophages play an important role limiting bacterial replication but can be overwhelmed with large inocula.

## 1 Introduction

*S. aureus* is a major cause of both community-acquired and hospital-acquired infections, causing a broad spectrum of disease ranging from skin and soft tissue infections to bacteraemia and infection of prosthetic devices (Cole et al., 2014). The pathogen contributes significantly to infection-related mortality and health-associated costs (de Kraker et al., 2011). Part of its success stems from a range of pathogen adaptations that subvert host defence (Cole et al., 2014). In addition it is resistant to a range of antimicrobials and virulent methicillin resistant strains of *S. aureus* (MRSA) have become a major health problem in many settings (DeLeo et al., 2010).

Macrophages are the resident phagocytes in tissues and play critical roles in host defense as the first professional phagocyte to encounter bacteria at sites of infection (Dockrell et al., 2003). Traditionally *S. aureus* has been classified as an extracellular bacterium that is readily phagocytosed and killed by phagocytes. Tissue macrophages are sufficient to clear *S. aureus* in murine models of pulmonary infection where mice are rendered neutropenic (Rehm et al., 1980), illustrating the potential role they can play in initial pathogen control at sites of infection. *S. aureus* is efficiently phagocytosed by macrophages (Jonsson et al., 1985). It is well established that *S. aureus* can avoid a range of innate immune responses from recognition to intracellular killing (Rooijakkers et al., 2006, Richardson et al., 2008) and this aids pathogenicity. In particular *S. aureus* employs several adaptations to resist oxidative stress and other microbicidal strategies utilised by phagocytes (Cole et al., 2014). We have recently demonstrated that differentiated macrophages, that model tissue macrophages such as the alveolar macrophage resident in the lung, although competent for bacterial clearance, have a finite capacity for intracellular killing, which is the rate limiting step in pathogen clearance (Jubrail et al., 2015).

Mechanistic mathematical models can be a vital tool in informing our understanding of complex biological systems. A number of mathematical models of the interactions between pathogens and host immune responses have been developed in recent years, with bacterial pathogens studied including *Mycobacterium tuberculosis* (Marino and Kirschner, 2004, Kischner and Marino, 2005, Warrender et al., 2006), *Bacillus anthracis* (Kumar et al., 2008, Day et al., 2011) and *Streptococcus pneumoniae* (Smith et al., Mochan et al.). In the context of macrophage function TB models are particularly relevant. TB is an intracellular pathogen that requires a competent macrophage-mediated immunological response for effective control of mycobacterial growth. Modelling approaches have shown that critical factors that influence pathogen growth include the recruitment and activation of macrophages, regulation of macrophage responses by cytokine signalling, and the recruitment of additional levels of the immune response that complement macrophage microbicidal strategies, such as T-cells and neutrophils (Kischner and Marino, 2005, Warrender et al., 2006, Tattevin et al., 2009). This has further refined understanding of the important roles macrophages play in this infection.

While there are a number of mathematical models addressing the dynamics of other bacterial and viral infections there is not currently a mathematical model describing the macrophage response to *S. aureus*. In part, this is because there has never been an extensive study exploring the kinetics of phagocytosis or intracellular killing of *S. aureus* by differentiated macrophages that could be used to develop a model for *S. aureus* infection. We have recently performed this kinetic analysis using differentiated macrophages, with key findings validated in primary human cells (Jubrail et al., 2015), and now use this data to develop a novel mathematical model. The model describes the extracellular and intracellular phases of *S. aureus* and their interaction with macrophages. These results provide important insights into how macrophages respond to *S. aureus*.

## 2 Methods

### 2.1 Experimental methods

#### 2.1.1 Macrophage phagocytosis

Experimental data was obtained using a differentiated tissue macrophage cell line THP-1 exposed to the Newman strain of *S. aureus* (Jubrail et al., 2015). Importantly the model uses a cell line whose differentiation status has been confirmed as replicating that of primary differentiated tissue macrophages (Daigneault et al., 2010) and findings have been validated *in vitro* with primary human monocyte-derived macrophages (hMDMs) and *in vivo* in murine alveolar macrophages. They have also been confirmed for the SH1000 strain and for the USA300 JE2 strain, a representative strain of community acquired MRSA, thus enhancing confidence in the broad relevance of the findings (Jubrail et al., 2015). Live THP-1 differentiated macrophages and paraformaldehyde fixed macrophages were cultured with *S aureus* Newman strain at a range of MOIs (multiplicity of infection) for up to 9 hours. Alternatively *S. aureus* Newman strain was cultured in the absence of macrophages for the same time course. At each time point, supernatants were plated onto blood agar for estimation of extracellular bacterial numbers using surface viability counts to determine the colony forming units (CFU). Extracellular bacteria were then killed with lysostaphin and cells lysed using 1% saponin to allow estimation of intracellular CFU (Jubrail et al., 2015).

#### 2.1.2 Macrophage killing

Macrophages were cultured with *S aureus* Newman strain at an MOI 5 for 6 hours and killing assays were performed as previously described (Jubrail et al., 2015). Extracellular bacteria were then killed with lysostaphin and some cultures treated with 1% saponin and lysed for intracellular CFU quantification. Remaining cultures were maintained in low dose lysostaphin for 0.5-4 hours and lysed at each time point for estimation of intracellular CFU. Results obtained with the high dose lysostaphin ‘pulse’ followed by the low dose lysostaphin ‘chase’ were verified using a similar approach with antimicrobials that selectively target the extracellular population but not intracellular bacteria, using a protocol employing gentamicin ‘pulse’ and vancomycin ‘chase’ to kill extracellular bacteria, to exclude any artefacts inherent in the use of lysostaphin (Jubrail et al., 2015). This data informs our selection of killing functions in the model, as described below.

Data for extracellular and intracellular bacteria density is presented for four different MOIs (0.05, 0.5, 1 and 5) with three replicates in each case. Active macrophage numbers (i.e. macrophages containing bacteria) are also plotted. These can be seen as the dots in Fig. 3. Here, we note two key elements of the data that the model will need to capture. Firstly, the extracellular bacteria densities remain low at low MOIs for much longer than at high MOIs (note these plots show logged numbers, so this is not merely an artefact of exponential growth). Secondly, the intracellular numbers appear to take-off at later time points at the higher MOIs but not the lower MOIs.

#### 2.2 Mathematical model

We model the interaction between macrophages (with densities, *M_i_*) and *S. aureus* (with densities, *S_i_*) using a set of ordinary differential equations as described below. We base the model on observations from the *in vitro* experiments and use this data to fit the model. We describe the model in detail below. There are similarities between our model and those of previous bacteria-cell studies (for example those of *M. tuberculosis* (Marino and Kirschner, 2004, Kischner and Marino, 2005)), but here our model is set up to intentionally mimic our experimental results, since important biological differences between *M. tuberculosis* and *S. aureus* are known, and as such there are a number of key differences to previous models.

#### 2.3 Macrophage cell dynamics

**Fig. 1:**
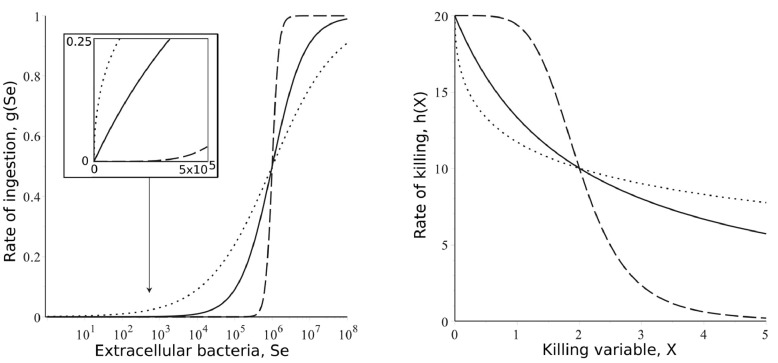
Examples of the ingestion (A) and killing (B) functions for different values of *p_β_* and *p_k_*. In each case solid lines are for $p_{i}=1$, dashed lines $p_{i}=4$ and dotted lines $p_{i}=0.4$. The inset in panel A shows the shape of the ingestion functions at low bacterial densities (here the horizontal axis is on a linear scale).

We assume that initially all macrophages are in a resting state, *M*_0_, and that to become competent for phagocytosis they need to become activated. The rate at which these cells reach this threshold for activation is an increasing, saturating function of extracellular bacteria, *S_e_*,

(1) $$f\left(S_{e}\right)=\alpha \frac{S_{e}^{p_{\alpha }}}{S_{e}^{p_{\alpha }}+{c_{\alpha }}^{p_{\alpha }}}$$

where *c_α_* is a threshold density at which the activation rate is *α*/2, and the power *p_α_* controls the shape of the function (c.f. Fig. 1a). In particular, if $p_{\alpha }=1$ then for low bacterial densities the increase in ingestion is linear (solid line, Fig. 1a, inset), while for *p_α_* > 1 at low densities there is very little ingestion (dashed line, Fig. 1a, inset) and for *p_α_* < 1 at low densities there is very high ingestion (dotted line, Fig. 1a, inset). We refer to those functions where *p_α_* ≤ 1 as ‘non-sigmoidal’, and those where *p_α_* > 1 as ‘sigmoidal’, due to their shapes (see Fig. 1a).

Activated cells become primed cells, *M_P_*, which are then able to ingest. The rate of ingestion is similarly given by an increasing, saturating function of extracellular bacteria, *S_e_*,

(2) $$g\left(S_{e}\right)=\beta \frac{S_{e}^{p_{\beta }}}{S_{e}^{p_{\beta }}+{c_{\beta }}^{p_{\beta }}}$$

where *c_β_* gives the density of *S_e_* at which the ingestion rate is $\frac{\beta }{2}$, and the power *p_β_* controls the shape of the function, in much the same way as the activation function above (Fig. 1a). Such a function has been used in the ecological literature for many years to represent predation, which is a similar process to ingestion here, as well as in previous bacteria-cell models (though these tend to assume $p_{\beta }=1$ (Marino and Kirschner, 2004, Kischner and Marino, 2005)) . Once cells have ingested bacteria we define them as active cells, *M_a_*, where they can continue to ingest. We do not differentiate between cells that have ingested but killed all of their bacteria and those that still contain viable bacteria. Functionally these cells behave the same, since we assume that once a cell ingests it will continue to do so as long as there are extracellular bacteria. (As the experimental *in vitro* data is derived from a situation where the extracellular bacteria out-compete the macrophage the situation where all bacteria are cleared is unlikely to arise.) Indeed, functionally the primed (*M_p_*) and active (*M_a_*) are very similar, but we require this differentiation both for the data fitting (since we only have data for active macrophages) and for certain implementations of killing (see below). We note that since we assume no production or decay of macrophages, verified from the experimental data, the total cell population $\bar{M}=\sum_{j\epsilon \{0,p,a\}}M_{j}$ is constant, meaning one of the equations can be neglected from the model.

#### 2.4 *S*. aureus dynamics

Initially all bacteria are extracellular, *S_e_*, with replication rate *r*. We assume that there is some maximum density (carrying capacity), *K*, for the bacteria in the medium, with classical logistic (logarithmic) growth in the absence of macrophages. During model fitting, below, a generalized logistic model was also tested but the power was always predicted to be near to 1, suggesting the standard logistic model is a reasonable parsimonious choice. Extracellular bacteria can be ingested by both primed and active cells.

When first ingested the intra-cellular *S. aureus* are still viable as *S_i_* bacteria. The active cells then kill the bacteria through phagocytosis-associated killing mechanisms and the bacteria become killed, *S_k_*. However, based on observations with neutrophils that phagocytosis activates the nicotinamide adenine dinucleotide phosphate oxidase (NADPH oxidase/ NOX2) system which is linked to a number of microbicidal mechanisms of rapid bacterial killing (DeLeo et al., 1999), and our own observations of the kinetics of intracellular *S. aureus* killing in macrophages, which suggest there is a phase of rapid killing immediately after phagocytosis followed by a gradual rate of later decline in bacterial viability (Jubrail et al., 2015), we assume that the ability of cells to kill the intra-cellular bacteria is lost over time. The cause of this loss in killing is not clear, but is likely due to exhausting a combination of bioenergetic demands (phagocytosis, killing, etc). Here we compare three different model assumptions. In model 1 we assume that the loss is strictly due to macrophages using up key resources during phagocytosis. In this case the rate of killing is a decreasing function of the average number of both viable and non-viable intra-cellular bacteria per active cell, $\left(S_{i}+S_{k}\right)/M_{a}$. In model 2 we assume that the loss is due to using up resources during killing, meaning the rate of killing is a decreasing function of the average number of only non-viable intra-cellular bacteria per active cell (i.e., those killed), *S_k_*/*M_a_*. Finally in model 3 we assume that the loss is due to the down-regulation of killing by macrophages as the infection density increases (for example, to maintain resources for phagocytosis). In this case we assume that killing is a decreasing function of the extra-cellular bacteria density, *S_e_*. As we say, in reality we expect a combination of these processes is involved, but these model assumptions may guide us in which processes are key. For each assumption, this equation can be given by,

(3) $$h\left(X\right)=k\left(1-\frac{X^{p_{k}}}{X^{p_{k}}+{c_{k}}^{p_{k}}}\right)$$

where $X\in [\left(S_{i}+S_{k}\right)/M_{a},\frac{S_{k}}{M_{a}},\;S_{e}]$ depending on the model (Fig. 1b). If $p_{k}=1$, the initial drop in killing is linear with *S. aureus* density. If *p_k_* > 1, killing initially remains high before a threshold effect and a sharp drop in killing. If *p_k_* < 1, then killing drops off quickly before saturating.

The full system of equations is given below. See Table 2 for a list of parameter definitions and a schematic of the model in ESM.

(4) $$\frac{dM_{P}}{dt}=\alpha M_{0}\left(\frac{S_{e}^{p_{\alpha }}}{S_{e}^{p_{\alpha }}+{c_{\alpha }}^{p_{\alpha }}}\right)-\beta M_{P}\left(\frac{S_{e}^{p_{\beta }}}{S_{e}^{p_{\beta }}+{c_{\beta }}^{p_{\beta }}}\right)$$

(5) $$\frac{dM_{a}}{\mathrm{dt}}=\beta M_{P}\left(\frac{S_{e}^{p_{\beta }}}{S_{e}^{p_{\beta }}+{c_{\beta }}^{p_{\beta }}}\right)$$

(6) $$\frac{dS_{e}}{dt}=rS_{e}\left(1-\frac{S_{e}}{K}\right)-\beta \left(M_{P}+M_{a}\right)\left(\frac{S_{e}^{p_{\beta }}}{S_{e}^{p_{\beta }}+{c_{\beta }}^{p_{\beta }}}\right)$$

(7) $$\frac{dS_{i}}{dt}=\beta \left(M_{P}+M_{a}\right)\left(\frac{S_{e}^{p_{\beta }}}{S_{e}^{p_{\beta }}+{c_{\beta }}^{p_{\beta }}}\right)-kS_{i}\left(1-\frac{X^{p_{k}}}{X^{p_{k}}+{c_{k}}^{p_{k}}}\right)$$

(8) $$\frac{dS_{k}}{dt}=kS_{i}\left(1-\frac{X^{p_{k}}}{X^{p_{k}}+{c_{k}}^{p_{k}}}\right)$$

#### 2.5 Model Fitting

The model is fitted to the experimental data using a rejection Approximate Bayesian Computation (ABC) approach Beaumont, 2010, Csill ´ery et al., 2010). Such a Bayesian approach is advantageous since it incorporates variation/uncertainty in the parameter estimates. Given that the experimental data itself reveals variation of as much as an order of 10, accounting for this variation is clearly important. Values for each parameter in the model are randomly sampled from uniform prior distributions with defined upper and lower limits chosen after preliminary runs. The model is then run using the ode45 solver in MATLAB and the sum of squares error calculated between the logged model output and logged data, summed over the intra- and extra-cellular densities at the four MOIs. Macrophage data is not included in the model fitting as it was gathered from a separate experiment, but is used as a comparison of models at a later stage. If the error is less than some chosen value, ɛ, the parameter set is accepted and stored, if it is greater than the value it is rejected. As ɛ becomes smaller the parameters should approach the ‘true’ values. 100,000 runs are performed for each model. This results in posterior distributions for every parameter of those sets kept for each model. Comparisons of the three models tested (where limitation in killing depends on ((1) average number of bacteria ingested, (2) average number of bacteria killed or (3) extracellular bacteria densities) are performed firstly by computing Bayes factors given by the ratio of acceptance rates for a given ɛ, and secondly by computing Akaike Information Criteria (AIC) for the (mean) least-squares error when fitted against the macrophage data.

An additional model assumption was tested in which for the first half hour there is neither bacterial growth nor ingestion, but this was found to produce relatively poor model fits.

## 3 Results

Table 1 presents a comparison of the performance of the three model variants tested (see Table 2 for the maxima and minima of the uniform priors). It is immediately clear that the least successful model is model 1 (killing is a function of the average number of bacteria ingested, $\left(S_{i}+S_{k}\right)/M_{a}$), with both a much higher minimum error and far fewer numbers of runs falling below arbitrarily chosen cut-offs than the other two models. Of the remaining assumptions, model 3 (killing is limited by extracellular density, *S_e_*) receives the stronger support. Comparing model 3 with model 2 (limitation through number of bacteria killed) the Bayes factor for the given values of epsilon are 6.75 (ε=8), 1.66 (ε=9) and 1.37 (ε=10). This indicates some positive, but not strong, support for selecting the model with limitation due to extracellular bacteria over the model with limitation due to killed bacteria (Kass and Raftery, 1995), with increasing support as the threshold for acceptance is reduced. Further investigation revealed some sensitivity to the chosen bounds of the priors, in particular the rate of priming, *α*. When this is constrained to a lower maximum the Bayes factor for model 3 vs model 2 improves dramatically due to reduced success of model 2 (with a maximum of *α*=10 the acceptances for ε=9 become 128 and 5 respectively, giving a Bayes factor of 25.6, indicating strong support for model 3). Additionally we compared the performance of each of these two models at fitting the as-yet unfitted macrophage data. Running 100 simulations with all parameter values chosen from the accepted posterior distributions (ε=9)of the two models, the average sum of squares error for model 3 is 51.14 and for model 2 is 104.12. Treating these averages as model runs, this yields a relative AIC (7 data points and 10 parameters) for model 2 of 0.08, again indicating reasonable but not overwhelming support for model 3 as the better fit. We suggest this unintuitive result indicates that killing is limited by a combination of bioenergetic processes, and that the extracellular density is providing the best ‘snapshot’ of the demands on the macrophages, with the non-negligible support for the limitation by killing model suggesting this may be a key process. For the remainder of the study we focus on the model with limitation through extracellular density.

**Table 1:** Details of model fitting from 10,000 runs with parameter values chosen from uniform distributions with maximum and minimum values as shown by the histograms in Fig. 2. This shows the minimum sum-of-squared error returned under each killing assumption, and the numbers of runs under certain error thresholds.

Killing assumption	Min. ϵ	Runs with ϵ <8	Runs with ϵ <9	Runs with ϵ <10
Model 3 S_e_	6.639	27	141	388
Model 2 *S_k_*/*M_a_*	7.761	4	85	284
Model 1 $\left(S_{i}+S_{k}\right)/M_{a}$	8.388	0	3	11

**Table 2:** Parameter descriptions, their mean values from model fitting with ε=9 and the range of values in the uniform prior distribution.

Parameter	Definition	Mean posterior value	Prior range
*α*	Basic priming rate of *M*_0_ cells	12.229	0.5-25
*c_α_*	Threshold density of priming function	2.223 × 10^4^	$10^{2}-10^{6}$
*p_α_*	Power (slope) of priming function	4.708	0-10
*β*	Basic ingestion rate of cells	3.232	0.5-8.5
*c_β_*	Threshold density of ingestion function	3.776 × 10^8^	$10^{2.5}-10^{9.5}$
*p_β_*	Power (slope) of ingestion function	0.557	0-2
*r*	Basic growth rate of S_e_ bacteria	0.571	0.4-0.8
*k*	Basic killing rate of cells	13.685	0-20
*c_k_*	Threshold density of killing function	2.159 × 10^7^	$10^{5.5}-10^{8.5}$
*p_k_*	Power (slope) of killing function	4.934	0-10

**Fig. 2:**
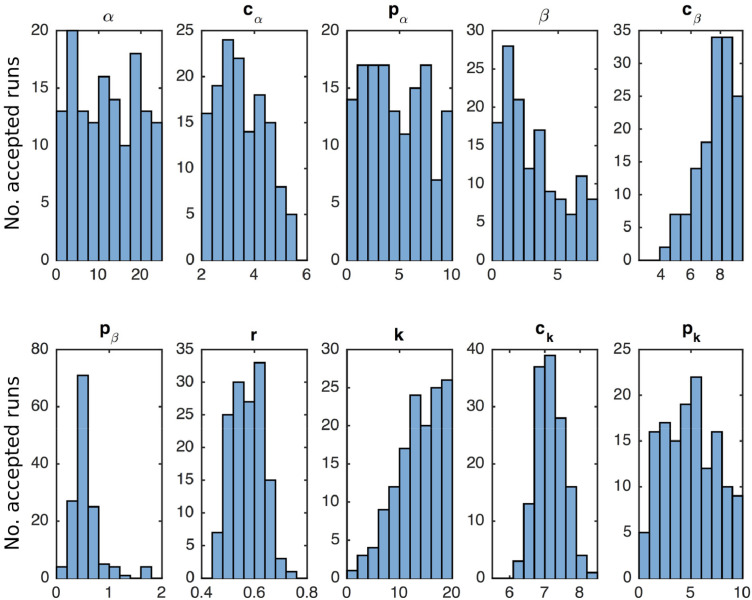
Posterior distributions for parameters in (4), (5), (6), (7), (8) from Approximate Bayesian Computation, with a sum of squares error threshold of 9 after 100,000 model runs. For each parameter 10 equally sized bins between the minimum and maximum of the uniform priors are used.

Fig. 2 shows the posterior distributions of the parameter values for the model with killing linked to extracellular density where ε=9. Note that the three threshold densities are plotted as logarithms. A number of the parameters present as normal distributions (particularly *p_β_*, *r*, *c_k_*), and others with clear indications that the parameter is ‘low’ or ‘high’ (e.g. *c_α_*, *β*, *c_β_*, *k*), giving us some confidence in the value of these parameters and their importance for the model. Other parameters yield rather uninformative posterior distributions, for example the power in the killing function, *p_k_*, is likely to be greater than 1 (and is thus sigmoidal) but could take almost any positive value and still return good fits. In contrast it is notable that the power in the ingestion function, *p_β_*, is very likely to be less than one and thus non-sigmoidal. This provides a clear contrast to many previous models of bacteria-cell dynamics that assume a power of unity (Marino and Kirschner, 2004, Kischner and Marino, 2005). Also, the threshold density for priming, *c_α_*, appears to be less than 10^5^, potentially with a high saturation constant, *p_α_*, suggesting that at very low MOIs few macrophages are primed at early time points, but at higher MOIs priming in response to bacteria happens rapidly.

**Fig. 3:**
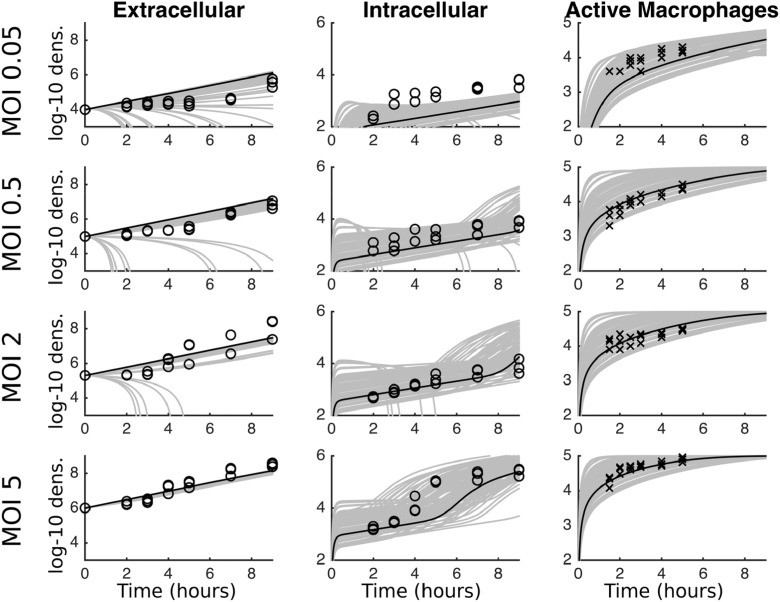
Experimental data (circles for bacteria data, crosses for macrophage data) and mathematical model fits for four MOIs. In each case the x-axis represents time in hours, and the y-axes are the log-10 densities. The black solid line gives the mean values from the posterior distributions in Fig. 2. The grey lines show 100 runs with the threshold values for ingestion, *c_β_*, and killing, *c_k_* chosen from the respective posterior distributions.

**Fig. 4:**
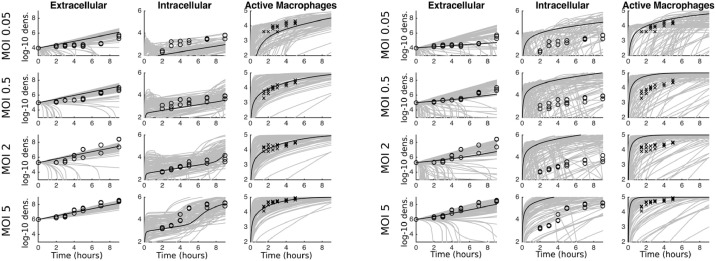
Similarly to Fig. 3**,** experimental data (circles for bacteria data, crosses for macrophage data) and mathematical model fits for four MOIs. In (a) all parameters are drawn from the posterior distributions in Fig. 2. In (b) all parameters are drawn from the prior distributions as in Table 2.

Fig. 3 plots the experimental data (circles for fitted data, crosses for unfitted data) against 100 runs where two parameters – the threshold values for ingestion, *c_β_*, and killing, *c_k_* – were drawn at random from the posterior distribution (grey lines) and all other parameters took the mean posterior value (see Fig. 2 and Table 2). One run using the mean values for all parameters from the posterior distributions is also highlighted (black line) (we acknowledge that the mean value is not necessarily representative of certain parameter values, but is a parsimonious choice for producing indicative results). Focusing on the mean (black solid line), the fit against the data is reasonable with two clear discrepancies: under-prediction of the number of intracellular bacteria at the lowest MOI, and a later acceleration of intracellular bacteria at the highest MOI. However, varying just the two parameters appears to account for almost all of the variation in the data, and in particular demonstrates the relatively slow extracellular dynamics at low MOIs and the take-off of intracellular numbers at the highest MOI. These two parameters, *c_β_* and *c_k_* were chosen as parameters that are unlikely to have ‘pre-determined’ fixed values and variability should be expected. Fig. 4a again shows 100 simulation runs but now with all parameters drawn from the posterior distribution, and now covering all variation in the data. Overall we suggest that there is some variability in many of the biological processes, and especially the ingestion and killing thresholds. Indeed, it is noticeable that we see variation within the three experimental data replicates in each MOI, especially at the higher doses. The existence of this variability lends weight to our selection of a Bayesian model fitting routine, which inherently captures the heterogeneity.

We conduct predictive checks of the model under both the prior and posterior parameter distributions to consider how well the fitted model is capturing the data (Maclaren et al., 2017). Fig. 4b is identical in its production to Fig. 4a except that here we have used the prior distributions for the parameter values. It is clear graphically that the fitted model performs significantly better at capturing the data. Moreover we can again use the unfitted macrophage data as an additional data set to measure the least-squares error as 100 full sets of parameter values are drawn at random from the posterior and prior distributions. Above we had found the posterior error had a mean of 51.14. For the prior distribution the mean error is 166.84, leading to a relative AIC of 0.016. Again, this gives a clear indication that the fitted model is better capturing the behavior of the data.

### 3.1 Mathematical analysis

We now perform a more formal mathematical analysis, informed by the model fitting above, to explore the system's behaviour. In particular, the behavior of the system (4)–(8) depends critically on the form of the ingestion function with different qualitative outcomes for non-sigmoidal and sigmoidal functions. Given the clear prediction that ingestion is non-sigmoidal (*p_β_* ≤ 1) from the model fitting, we focus only on this case.

Eqs. (4)–(6) for the macrophage and extracellular bacteria densities form a closed system that can be solved. For non-sigmoidal ingestion there are two biologically-relevant (that can be both stable and positive) equilibria. The first is an *infection-free* case ($S_{e}=0,\;M_{p}=Y-X,\;M_{a}=X$) where the macrophages have successfully eradicated the infection, taking *X* active cells to do so (with $M_{0}=\bar{M}-Y$ un-primed cells remaining). Since there are no extracellular bacteria we would also expect the intracellular numbers to eventually decrease to zero, though this may be a very slow process if the intra-cellular densities had got too high before the infection was eradicated. For the special case of $p_{\beta }=1$ this equilibrium is stable when,

$$r<\frac{\beta \bar{M}}{c_{\beta }},$$

in other words when the growth rate of the bacteria is lower than the ingestion rate of the macrophages at low bacteria densities. Based on the parameter estimates above this would require very high cell densities (~10^7^) to be possible. More generally, for *p_β_* < 1, the infection-free equilibrium is always locally stable since at very low bacterial densities we always have $\frac{dS_{e}}{dt}<0$ due to the relatively high ingestion rates at low bacterial densities, though the basin of attraction (in terms of *S_e_* densities) for this equilibrium shrinks rapidly as $\bar{M}\to 0$ (see Fig. 6 below).

The second case is an *endemic* quasi-equilibrium, which for the special case of $p_{\beta }=1$ occurs at ($S_{e}=\left(K-c_{\beta }+\sqrt{{\left(K+c_{\beta }\right)}^{2}-4K\bar{\frac{M}{r}}}\right)/2,\;M_{p}=0,\;M_{a}=\bar{M}$). The case when *p_β_* < 1 will be qualitatively similar (as can be seen by plotting the two terms of equation (6) and considering how the two curves will cross). Here all macrophages contain bacteria and the extracellular numbers have settled to an equilibrium that is a small amount below their carrying capacity. The percentage reduction is proportional to $\sqrt{\bar{M}/K}$, which would mean a very small reduction given the densities used in the accompanying experimental work ($\sqrt{10^{5}/10^{9}}=1\%\;$reduction from *K*). While this final reduction may be minimal, the impact of the macrophages on the initial growth is starkly illustrated by considering the *per-capita* growth rate of extracellular bacteria in equation (6). This is given by,

(9) $$\rho =r\left(1-\frac{S_{e}}{K}\right)-\beta \left(M_{P}+M_{a}\right)\left(\frac{S_{e}^{p_{\beta }-1}}{S_{e}^{p_{\beta }}+{c_{\beta }}^{p_{\beta }}}\right).$$

**Fig. 5:**
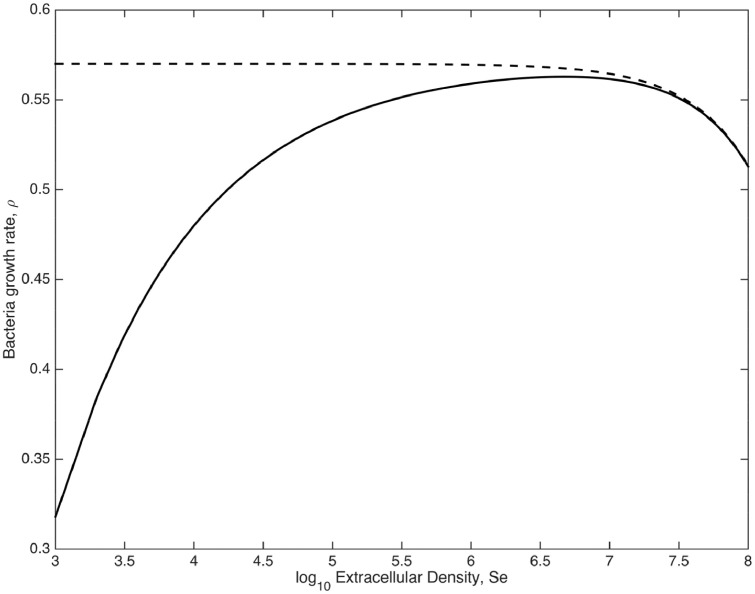
The per-capita growth rate, *ρ*, Eq. (9), of extracellular bacteria, with values set as the mean from the posterior distributions in Fig. 2. In particular $p_{\beta }=0.557$ (solid line). For comparison $p_{\beta }=1$ (dashed) is also shown.

Under the assumption that all macrophages are primed (such that $M_{P}+M_{a}=\bar{M})$, we can compare the case where $p_{\beta }=1$ (dashed) with the mean value predicted from the model fitting $p_{\beta }=0.557$ (solid) in Fig. 5. It is striking that at lower bacterial densities, under the predicted shape of the ingestion function, *g*(*S_e_*) (equation (2)), the macrophages’ response have a considerable impact on extracellular growth, thus causing a significant delay in the bacteria approaching its final density.

We term this second case considered above a quasi-equilibrium since the number of intracellular bacteria will in fact continue to increase by Eq. (7). The quasi-equilibrium only exists up to a certain value of $\bar{M}$, and for higher values only the infection-free state is feasible. However, even for lower values of $\bar{M}$, where both equilibria are feasible, there is a region of bistability in the system as the stability condition is distinct from that of the infection-free case, which for the special case of $p_{\beta }=1$ is given by,

$$r>\frac{\beta \bar{M}c_{\beta }K}{{\left(S_{e}^{*}+c_{\beta }\right)}^{2}\left(K-2S_{e}^{*}\right)}.$$

**Fig. 6:**
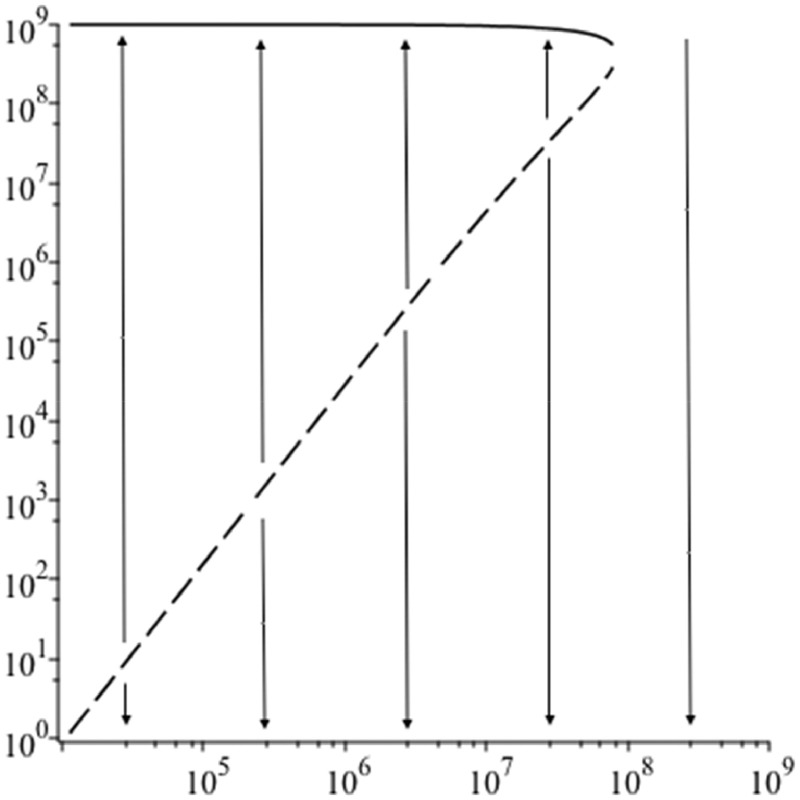
**–** Location and stability of extracellular bacteria equilibria for varying macrophage numbers, $\bar{M}$. Solid line gives the stable equilibrium, the dashed line the unstable equilibrium. Arrows show the basin of attraction. Parameter values are the means of the posterior distributions in Fig. 2 as given in Table 2.

For the more general case of *p_β_* < 1, as we have noted, the infection-free equilibrium is always locally stable, and this bistability exists for the whole range of $\bar{M}$ for which the endemic quasi-equilbirium is feasible. This can be seen clearly by plotting the equilibrium values for *S_e_* as the total cell density is varied as in Fig. 6 (using the mean parameter values from the posterior distributions in Fig. 1). At low values of $\bar{M}$ the basin of attraction for the endemic quasi-equilibrium is very large and even small doses of bacteria will grow to close to the carrying capacity. Indeed, for the population sizes used here (10^5^), a bacterial dose of 100 (i.e. an MOI of 0.001) would see the bacteria grow. For very high values of $\bar{M}$ the bacteria will always be eradicated. For intermediate densities, however, the outcome depends on the initial density of bacteria, with full control possible for realistic *S. aureus* doses if the macrophage population is large enough.

## 4 Discussion

We find that at low doses of *S. aureus* infection macrophages delay exponential growth of the bacteria for a number of hours, buying the host time to recruit other inflammatory cells to control infection. At higher doses the response can be overwhelmed such that there is almost no control. We identified bistabilities in the system such that a large and fast enough macrophage response may be able to significantly limit the final size of the infection provided the dose is low. We also found that killing is the key limiting step in the macrophage response to all infections, but that ingestion fails to keep pace with bacterial growth at high bacterial densities. Our model provides a good fit to experimental data and provides important insights into the early course of *S. aureus* infections.

Previously there has not been robust experimental kinetic data involving differentiated macrophages on which to base model parameters. A great strength of this model is that it is informed by data with a differentiated macrophage cell line that replicates the phagocytic and microbicidal response of differentiated tissue macrophages (Daigneault et al., 2010). Moreover the experimental data has been validated for primary human macrophages and in the clinically relevant JE2 strain (Jubrail et al., 2015). This makes the data relevant to major clinical strains that are highly prevalent, such as USA300. These play a major role in current community-acquired MRSA infections and are a major cause of skin and soft tissue infection (DeLeo et al., 1999). Based on this experimental data our model has shown that macrophages may not fully clear an *S. aureus* infection even at relatively low initial doses. The growth rate of the bacteria generally outstrips the ingestion rate of the macrophages for parameter values estimated from experimental data. The macrophage response will reduce the final size of the extracellular bacterial population, but only by a small percentage. However, we do see that at low doses the macrophages are able to delay exponential growth of the bacteria for a number of hours due to the relatively high ingestion rate at low bacterial densities. *In vivo* this may allow the immune system to ‘buy time’ while further immune cells, including neutrophils and T-cells, are recruited to the site of infection. This extended control of infection at lower doses could activate downstream signalling pathways in active macrophages leading to the recrutiment of monocytes and/or other immune cells to further help with infection control. Macrophages play important roles generating the cytokine networks required to recruit other immune cells during the immune response to Gram-positive bacteria such as *S. aureus* (Cole et al., 2014), so the extra time they buy during infection may be highly relevant for infection outcome. A clear area for future modelling is to incorporate the recruitment of such cells to give a more realistic model of an *in vivo* infection, similarly to models of *Mycobacterium* (Kischner and Marino, 2005) and *Streptococcus* (Smith et al.).

It is clear from our results that the killing ability of macrophages is finite and becomes overwhelmed with sustained killing. Interestingly, our Bayesian model fitting indicates that this limitation may be linked to the extracellular bacteria density. We suggest this result reflects that it is not individual processes related to intracellular numbers, such as ingestion or killing, but the cumulative effect of all of these that limits clearance. It may be that extracellular numbers are a better surrogate for the overall effect of intracellular burden since measures of viable or non-viable bacteria intracellularly represent only a ‘snapshot’ of overall interacellular burden and clearance. What is clear, however, is that after macrophages have been actively tackling relatively low numbers of bacteria the killing rate shows a significant drop-off to a state of almost no further killing. This fits with current knowledge from experimental studies of this system that loss of killing is a significant limiting step in macrophages’ response to *S. aureus* infection (Jubrail et al., 2015). Stimulating macrophages with the M1 cytokine interferon gamma (IFNγ) only modestly improved intracellular killing suggesting that failure of classical macrophage activation was not responsible and intracellular killing capabilities had been overwhelmed irrespective of classical activation (Jubrail et al., 2015). The ingestion ability of macrophages, on the contrary, showed no evidence of a decline, but the rate of ingestion did saturate at medium bacteria densities since cells cannot physically ingest more than a set number of bacteria in a certain time. Thus our model identifies that ingestion by macrophages is also an important limiting step as the size of infection grows. However, at low bacteria densities the model fitting suggests that the macrophages are rather efficient at ingesting and that full eradication of moderate inocula would be possible for large enough macrophage numbers. This emphasises the importance of the macrophage response being launched before the infection has grown too large. It would be interesting to consider the extent to which such dose-dependent responses are seen in other pathogens.

While we have based our model on *in vitro* experimental results, our model has suggested that complete eradication of low dose infections would be possible if far more macrophages were added, provided they were in the optimal polarisation status. This would have the effect of increasing the ingestion rate of the macrophage population. While this was not tested in our experimental set-up, *in vivo* it may be possible if recruitment of additional monocytes is fast enough. Monocytes have been shown to contribute to control of *S. aureus* in animal models (Veltrop et al., 2000) and recruitment of monocytes from the blood into the tissues and subsequent differentiation into macrophages could be beneficial for pathogen control. There is also evidence of local proliferation of alternatively activated macrophages within tissues under conditions where Th2 cytokines dominate (Jenkins et al., 2011, Jenkins et al., 2013), as would occur during conditions such as eczema and atopic dermatitis, that are associated with *S. aureus* colonisation (Gong et al., 2006). Thus one can envisage clinical scenarios where macrophage numbers responding to *S. aureus* could be supplemented either from monocyte-derived or tissue-derived populations to help control infection. However, it reamins unclear whether these alternative sources would enhance clearance or provide extra cells in which ingestion might promote a population of viable intracellular opulation of bacteria since ingestion without complete killing can favour escape from macrophage host defence by Gram-positive bacteria, as recently demonstrated for sub-populations of splenic macrophages that fail to clear ingested *S. pneumoniae*, promoting sepsis (Ercoli et al., 2018). Therefore the number of ‘active’ macrophages is likely to be a critical determinant of outcome. In this respect an increase in numbers of ‘active’ macrophages and the ability to prime cells for this function is likely to vary between individuals explaining some of the variation in ability to handle bacteria observed. This would be more likely than altering numbers of tissue macrophages at the site of infection in the initial period .With the exception of clinical stiuations such as radiation or inhalation of toxic fumes variation in tissue macrophage numbers is less likely to vary than the qualitative function of these cells (Cole et al., 2014).

An important finding from the model is that there is bistability between different states. This means that infections at low doses are much more easily controlled than those at high doses, highlighting the importance of a fast immune response to *S. aureus* infection. Moreover, the bistability means that simply calculating an “*R*_0_-like” bacteria-free stability condition is not enough to ensure control, and if the immune response, or indeed treatment, is delayed then control of the infection is much harder. An implication of this is that if the immune competence of the macrophage is altered control of *S. aureus* may be less successful. An example of this clinically could be the observation that *S. aureus* skin infections with virulent MRSA strains are more frequently encountered in people living with HIV infection (Popovich et al., 2010), a setting where macrophage function is altered (Collini et al., 2010). Variation in numbers of ‘active’ macrophages represents a plausible explanation to how the the bistability in the model we observed could lead to variation in disease outcome between patient groups.

The model fitting highlighted that there appears to be some variation in a number of the mechanistic rates, as a single parameter set failed to fit all of the experimental data well simultaneously, particularly of the timing of sudden increases in intracellular bacteria. Our approach of using Approximate Bayesian Computation allowed us to overcome this by including variability in the parameter estimates. In particular, allowing variation in the parameter values for the killing and ingestion thresholds allowed most of the variation in the data to be predicted, suggesting that these processes may not be homogenous or some biologically relevant terms may be missing from the model. As well as the future extensions already mentioned above, it may therefore also be important to explore the impact of macrophage heterogeneity. Tissue heterogeneity in macrophage populations is increasingly recognised both in terms of the variation between tissues (Gordon et al., 2014) and the range of phenotypes within a tissue (Laskin et al., 2001) and future work should explore this. There could also be signalling between macrophages allowing an exhausted macrophage to elicit a signal to a nearby macrophage which can pick up the released bacteria. The concept of direct and indirect paracrine effects in response to a pathogen are well characterized in infectious diseases and can be transmitted by cytokines or other signaling peptides produced by the host cells (Mosser and Edwards, 2010). These cytokine responses lead to paracrine effects on neighbouring cells that can enhance pathogen clearance. Again, models of other bacteria-cell interactions have included cytokine signaling (Kischner and Marino, 2005, Smith et al.) and would be an important extension to explore here.

In conclusion, we have shown that based on unique experimental data we do not expect macrophages to completely eradicate *S. aureus* infections on their own except at very low inocula, but that they will delay the exponential growth phase, buying time for recruitment of further immune cells. In addition supplementing numbers of activated macrophages can further enhance the macrophages’ contribution to the immune response and clearance of *S. aureus*. Thus despite *S. aureus* ability to subvert macrophage innate responses macrophages still make a major contribution to host defense against this pathogen.

## Author Contributions

AB helped design the study, carried out the mathematical analsyis and model fitting and drafted the manuscript; JJ carried out the experimental work, helped with the mathematical analysis and helped draft the manuscript; MB conceived of the study and helped draft the manuscript; DD conceived of the study, helped design the study and helped draft the manuscript; HM conceived of the study, helped design the study, helped carry out the experimental work and helped draft the manuscript.

## Data

The data is available from chapter 3 on the thesis found at http://etheses.whiterose.ac.uk/id/eprint/6636.

## Declaration of Competing Interest

We have no competing interests.

## Appendix Supplementary materials

Image, image 1
