# Supplementary figures and images for: A mathematical model shows macrophages delay Staphylococcus aureus replication, but limitations in microbicidal capacity restrict bacterial clearance
Source: J Theor Biol. 2020 Jul 21;497:110256. doi: 10.1016/j.jtbi.2020.110256 (PMC7262596; doi:10.1016/j.jtbi.2020.110256)

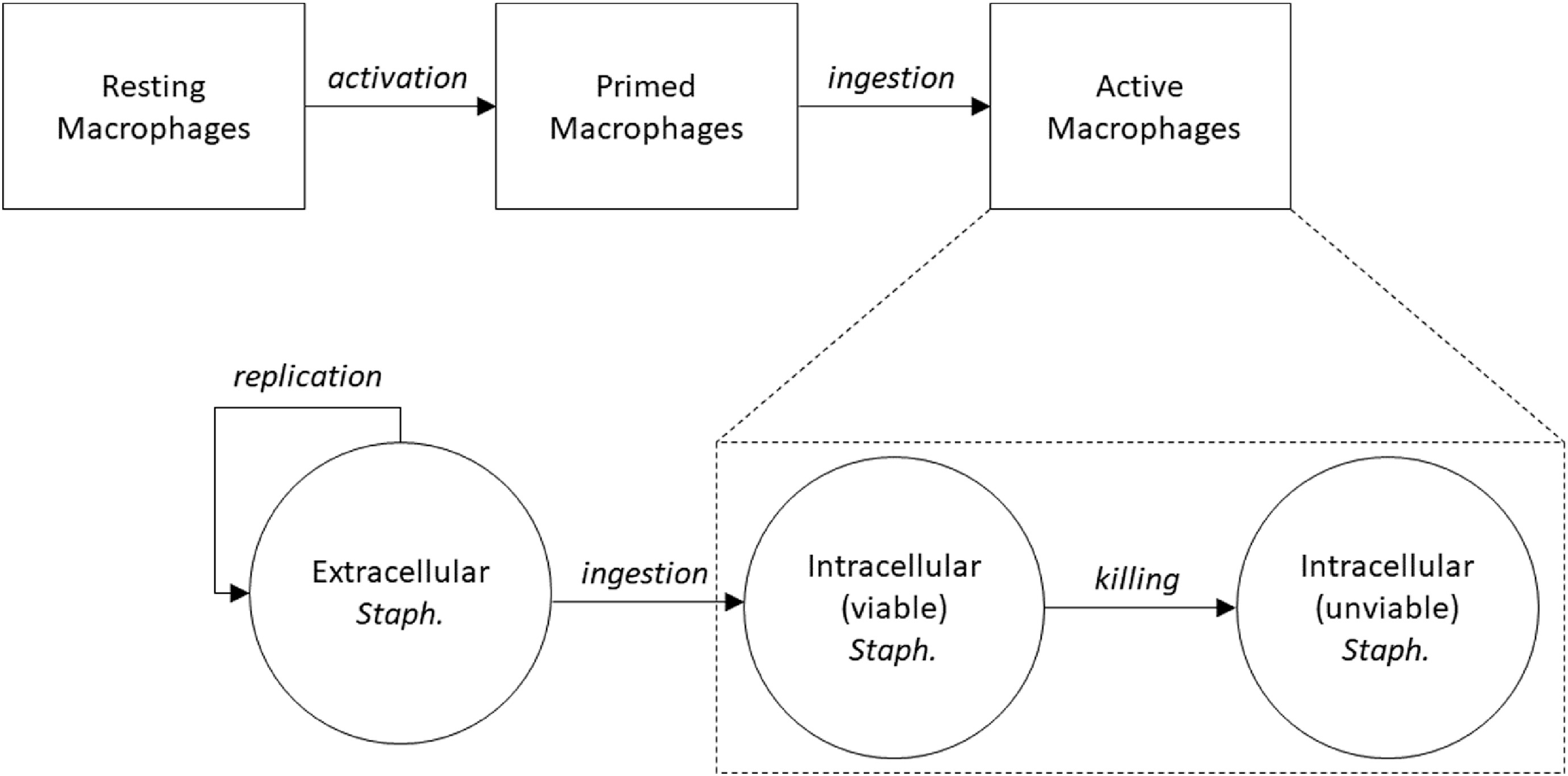

Supplement: Supplementary file 1 [file mmc1.jpg]
